# Formulation and In-Vitro Characterization of pH-Responsive Semi-Interpenetrating Polymer Network Hydrogels for Controlled Release of Ketorolac Tromethamine

**DOI:** 10.3390/gels7040167

**Published:** 2021-10-13

**Authors:** Muhammad Suhail, Yi-Han Hsieh, Yu-Fang Shao, Muhammad Usman Minhas, Pao-Chu Wu

**Affiliations:** 1 School of Pharmacy, Kaohsiung Medical University, Kaohsiung City 80708, Taiwan; suhailpharmacist26@gmail.com; 2 Department of Biomedical Science and Environmental Biology, Kaohsiung Medical University, Kaohsiung City 80708, Taiwan; irene93303@gmail.com (Y.-H.H.); irissshao@gmail.com (Y.-F.S.); 3 College of Pharmacy, University of Sargodha, Sargodha 40100, Pakistan; 4 Department of Medical Research, Kaohsiung Medical University Hospital, Kaohsiung 80708, Taiwan; 5 Drug Development and Value Creation Research Center, Kaohsiung Medical University, Kaohsiung 80708, Taiwan

**Keywords:** carbopol 934, sodium polystyrene sulfonate, ketorolac tromethamine, hydrogel

## Abstract

Ketorolac tromethamine is a non-steroidal anti-inflammatory drug used in the management of severe pain. The half-life of Ketorolac tromethamine is within the range of 2.5–4 h. Hence, repeated doses of Ketorolac tromethamine are needed in a day to maintain the therapeutic level. However, taking several doses of Ketorolac tromethamine in a day generates certain complications, such as acute renal failure and gastrointestinal ulceration. Therefore, a polymeric-controlled drug delivery system is needed that could prolong the release of Ketorolac tromethamine. Therefore, in the current study, pH-responsive carbopol 934/sodium polystyrene sulfonate-co-poly(acrylic acid) (CP/SpScPAA) hydrogels were developed by the free radical polymerization technique for the controlled release of Ketorolac tromethamine. Monomer acrylic acid was crosslinked with the polymers carbopol 934 and sodium polystyrene sulfonate by the cross-linker *N*’,*N*’-methylene bisacrylamide. Various studies were conducted to evaluate and assess the various parameters of the fabricated hydrogels. The compatibility of the constituents used in the preparation of hydrogels was confirmed by FTIR analysis, whereas the thermal stability of the unreacted polymers and developed hydrogels was analyzed by TGA and DSC, respectively. A smooth and porous surface was indicated by SEM. The crystallinity of carbopol 934, sodium polystyrene sulfonate, and the prepared hydrogels was evaluated by PXRD, which revealed a reduction in the crystallinity of reactants for the developed hydrogels. The pH sensitivity of the polymeric hydrogel networks was confirmed by dynamic swelling and in vitro release studies with two different pH media i.e., pH 1.2 and 7.4, respectively. Maximum swelling was exhibited at pH 7.4 compared to pH 1.2 and, likewise, a greater percent drug release was perceived at pH 7.4. Conclusively, we can demonstrate that the developed pH-sensitive hydrogel network could be employed as a suitable carrier for the controlled delivery of Ketorolac tromethamine.

## 1. Introduction

An ideal controlled drug delivery system (CDDS) is one that delivers the drug locally or systemically at a predetermined rate for a specified period of time. The main goals of CDDSs are to ensure safety and enhance drug efficacy with improved patient compliance [1]. They are involved in the delivery of drugs to a specific/target site in the body. Several methodologies and traditional disciplines are involved in controlled drug delivery as it is a broad field. CDDS also delivers effective therapy for an extended period of time while eliminating or decreasing most of the adverse effects concerned with other conventional drug therapies [2]. A drug delivery system is considered to be ideal if the system releases the drug in response to specific stimuli such as light, temperature, pH, or pressure [3]. Hydrogels are known as smart drug delivery systems [4]. Hydrogels are three-dimensional network systems with the capacity to imbibe a greater quantity of water due to the presence of physical or chemical crosslinkage of hydrophilic polymer chains [5,6]. Due to the chemical or physical cross linking, these systems are not dissolved by the respective medium [6,7]. Both physical and chemical crosslinking methods are used in the development of hydrogel formulations [8,9].

The current challenge for existing drugs is improvement of their safety efficacy ratio, which needs to be addressed rather than the development of novel drugs that involve high cost and time requirements. Low aqueous solubility, inadequate absorption along the gastrointestinal (GI) tract, degradation risk of drugs in the acidic milieu of the stomach, systematic side effects, and low permeability of the drugs in the upper GI tract, etc., are the factors that affect the efficacy of the drugs. Therefore, a polymeric system is needed to overcome all the aforementioned factors. Hence, hydrogels, especially stimuli-responsive hydrogels, are considered to be the suitable candidate for addressing the challenges of existing drugs. The most-studied hydrogels amongst stimuli-responsive hydrogels are the pH-sensitive hydrogels. Certain abrupt changes, such as collapsing and swelling, occur when stimuli-sensitive hydrogels are exposed to a particular stimulus leading to the volume phase transition. The size, shape, number of ionic groups, cross-linking density, and composition are the factors that determine the rate at which hydrogels respond. A decrease in crosslinking density leads to an increase in pore size and the number of ionic groups, which further increases the response rate of hydrogels [10]. pH-responsive hydrogels are used for the delivery of drugs to the specific site of the gastro-intestinal tract [11]. Khalid et al. (2017) prepared pH-responsive alginate-PVA-based semi-IPN hydrogels and reported high swelling and percent drug release at pH 7.4 compared to pH 1.2 [12]. Al-Tabakha et al. (2021) developed sericin-based hydrogels and reported maximum swelling and Acyclovir percent release for the developed hydrogels in a basic medium compared to an acidic medium [13]. Similarly, Kaleem et al. (2019) fabricated gelatin-based hydrogels for the colonic delivery of Oxaliplatin and demonstrated a pH-responsive nature of the fabricated hydrogels due to the presence of acrylic acid [14]. In a recent study, pH-responsive carbopol 934/sodium polystyrene sulfonate-co-poly(acrylic acid) hydrogels were formed. The incorporation of carbopol with sodium polystyrene sulfonate and acrylic acid in the presence of the crosslinker MBA enhanced the worth of the recent study more than the previously reported research work. Greater swelling and percent drug release were detected at pH 7.4 compared to pH 1.2. Similarly, a high gel fraction and maximum drug loading were observed for the reported hydrogels compared to previously report pH-responsive hydrogels. Similarly, high porosity and thermal stability were revealed by fabricated hydrogels compared to the previous reported research work. Hence, we can conclude that the developed pH-responsive hydrogels have the capability of prolonging the release of drugs for an extended period of time.

A vital role is played by different carbopol polymers in the fabrication of stimuli-responsive hydrogels because they make changes in their swelling behavior once exposed to external stimuli such as temperature [15], pH [3,9], an electric field, or light. Carbopol 934 (CP) is also recognized as a smart gel or environmentally responsive polymer [16,17]. In modern eras, researchers have focused on CP as it is understood as a suitable candidate for the development of various polymeric networks especially for controlled drug delivery systems. CP plays a key role in the delivery of a drug to the target area of the body [18]. Due to its pH-sensitive nature, high swelling and drug release are observed by CP in a basic medium in carbopol-based hydrogels [19]. Polystyrene sulfonate is a novel, noncytotoxic, antimicrobial, and polydisperse linear sulfonated polymer. It is highly soluble in water (>330 mg/mL) and aqueous alkaline solutions. The high molecular weight of polystyrene sulfonate is 500–800 kDa and its sodium salt is sodium polystyrene sulfonate (SpS), which is used for different purposes. Polystyrene sulfonate is non-mutagenic and has a very low oral toxicity (LD50 > 5 g/kg body weight in rats) [20]. Due to the high uptake of free ions, SpS exhibits more swelling at a specific pH, and is thus used commonly in the preparation of polymeric systems, mainly in hydrogels. Due to the existence of various functional groups, particularly SO_3_, the use of SpS is not limited to biomedical and pharmaceutical fields [21]. Acrylic acid (AA) is a water-soluble and pH-sensitive polymer [22,23,24]. Due to its pH-sensitive nature, AA is employed in stimuli-sensitive drug delivery systems, particularly in the development of pH-sensitive hydrogels. Like other pH-sensitive polymers, the swelling index of AA is greater at a basic pH, therefore maximum drug release is observed in a basic medium and thus used broadly in biomedical and pharmaceutical fields [12].

Ketorolac tromethamine (KT) is a non-steroidal anti-inflammatory drug (NSAID) prescribed for the management of severe pain, with low anti-inflammatory and high analgesic activity [25,26]. The reported results indicated the less-hepatic first-pass elimination of KT. The half-life of KT is in the range of 2.5–4 h. Thus, repeated doses of KT administration are required due to the short half-life in order to retain the therapeutic level. However, taking several doses of KT in a day leads to certain complications, including acute renal failure and gastrointestinal ulceration [27]. Recurrent usage of KT decreases the patient’s compliance. Therefore, in order to overcome the complications associated with repeated doses of KT and enhance patient compliance, a polymeric system is required for the controlled delivery of KT [28]. Hence, the most suitable polymeric system for the controlled delivery of KT is hydrogels, due to their high swelling capability, drug loading, controlled release, and low cytotoxicity. The crosslinking of CP, SpS, and AA enabled the developed hydrogels to prolong the release of KT for an extended period of time in a controlled way.

In the current study, the authors developed the CP/SpScPAA-based semi-interpenetrating polymer network (SIPN) hydrogels by the free radical polymerization technique for the controlled delivery of KT. The prepared hydrogels were subjected to a series of studies including FTIR, sol–gel analysis, polymer volume fraction, TGA, DSC, dynamic swelling, SEM, drug loading, in vitro drug release, kinetic modeling, percent porosity, and PXRD. The reported results indicate the controlled release of KT from the developed hydrogels.

## 2. Results and Discussion

### 2.1. Fourier Transform Infrared (FTIR) Spectroscopy

**Figure 1:**
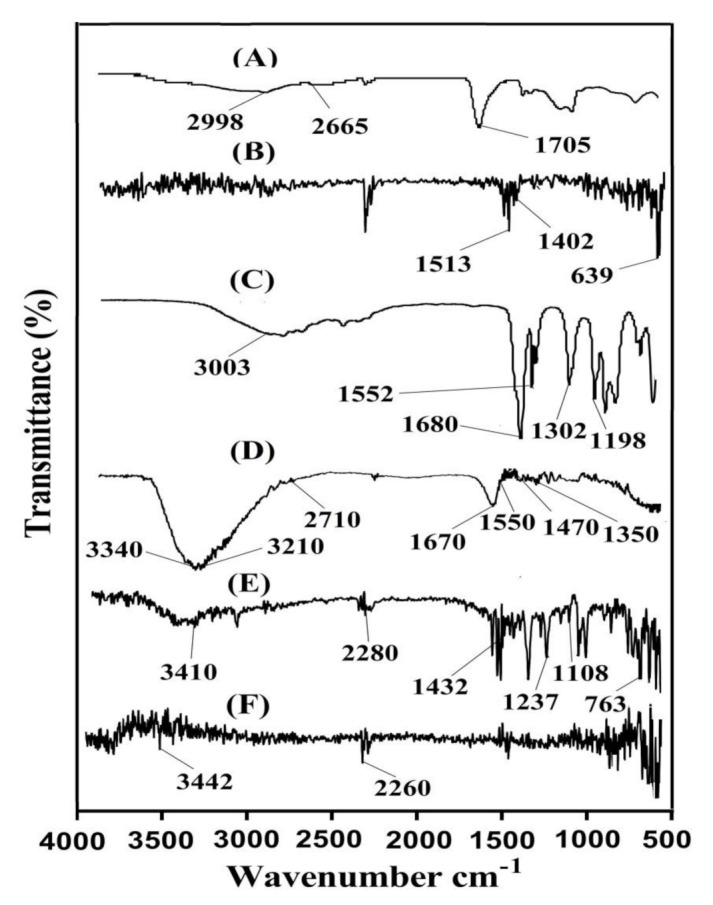
FTIR spectra of (**A**) CP, (**B**) SpS, (**C**) AA, (**D**) unloaded CP/SpScPAA hydrogels, (**E**) KT, and (**F**) drug-loaded CP/SpScPAA hydrogels.

FTIR spectroscopy was used for the structural evaluation of the fabricated hydrogel and its constituents. The FTIR spectrum of CP, SpS, AA, unloaded CP/SpScPAA hydrogels, KT, and drug-loaded CP/SpScPAA hydrogels is shown in Figure 1A–F. FTIR spectra of CP (Figure 1A) reveal characteristic peaks at 1705, 2665, and 2998 cm^−1^ corresponding to the stretching vibration of C=O, OH, and R–CH_2_, respectively [29,30,31]. FTIR spectra of SpS (Figure 1B) indicate peaks at 1402 and 1513 cm^−1^ revealing the symmetric and asymmetric vibration of the SO_3_ group. Similarly, the aromatic stretching vibration of C–H is assigned by a peak at 639 cm^−1^ [32]. Likewise, the distinct peaks of AA (Figure 1C) at 1198 and 1302 assign the stretching vibration of –C–C and C–O, whereas bands at 1680 and 1552 cm^−1^ indicate the stretching vibration of C=O and the bending of the carboxylic group (C=O), respectively. Along with this, the absorption peak at 3003 cm^−1^ is assigned to the stretching vibration of O–H [33]. The FTIR spectrum of unloaded CP/SpScPAA (Figure 1D) hydrogels indicates a modification in the functional group’s position of the CP, SpS, and AA due to the electrostatic interaction among them. The prominent CP bands at 2665 and 2998 cm^−1^ are altered to 2710 and 3210 cm^−1^ bands in unloaded CP/SpScPAA hydrogels. Similarly, the characteristic peaks of SpS and AA at 1402, 1513 cm^−1^, and 1302, 1680, 3003 cm^−1^ are modified to 1470, 1550 cm^−1^, and 1350, 1670, 3340 cm^−1^, respectively. Some peaks of CP, SpS, and AA disappeared while a few new peaks formed. The modification, formation, and disappearance of peaks indicate the change in the peak frequency of CP, SpS, and AA. Thus, this all shows the development of CP/SpScPAA hydrogels due to the grafting of AA on the backbone of CP and SpS. KT indicates FTIR spectra (Figure 1E) by peaks at 3410, 1432, 1237, and 1108 cm^−1^ corresponding to the stretching vibration of N–H and NH_2_, –C–N, C=O (diaryl ketone), and –OH, respectively. Similarly, the bending of C–H (Aromatic) is indicated by peaks at 2280 and 763 cm^−1^ [33,34,35,36]. A minor change is seen in the distinct peaks of the drug in the FTIR spectrum of drug-loaded CP/SpScPAA hydrogels (Figure 1F) due to the loading of the drug by the developed hydrogel networks. The prominent KT peaks at 3410 and 2280 cm^−1^ are slightly changed to 3442 and 2260 cm^−1^ in drug-loaded CP/SpScPAA hydrogels. The presence of drug peaks in the drug-loaded CP/SpScPAA hydrogels demonstrates the successful loading of the drug by fabricated hydrogels without any kind of interaction [37].

### 2.2. Sol-Gel Analysis

Sol-gel analysis is carried out for all formulations of fabricated CP/SpScPAA hydrogels. This study is conducted for the purpose of determining the un-crosslinked soluble (Sol) and crosslinked insoluble (Gel) fractions of the hydrogels. Hydrogel contents i.e., CP, SpS, and AA, affect the sol and gel fractions as shown in Table 1. An increase in gel fraction is observed with the increase in the composition of CP and SpS. Polymer plays an important role in the polymerization reaction. An increase in polymer composition causes the rapid generation of free radicals and high availability of reactive sites for the polymerization reaction. Furthermore, when two or more polymers are used in the development of hydrogels, an increase in the reactive sites for the monomer is observed with the increase in polymer composition. Hence, as the composition of both CP and SpS is increased, the gel fraction is increased due to the availability of a high number of reactive sites for the monomer. Therefore, we can conclude that the polymerization reaction among hydrogel contents will be higher if greater numbers of reactive sites are available, and vice versa. Khalid et al. (2018) prepared CS-co-poly(AMPS)-based hydrogels and reported that the gel fraction is increased as the composition of the polymer is increased [38], which further supports our study. Likewise, the gel fraction is increased as the composition of AA is increased [39,40]. Unlike the gel fraction, a decrease in the sol fraction is seen as the composition of CP, SpS, and AA increases because there is an inverse proportion between the sol and gel fraction [41].

### 2.3. Polymer Volume Fraction

The polymer volume fraction is analyzed for all formulations of the developed hydrogels at both pH 1.2 and 7.4 (Table 1), and a high polymer fraction is detected at pH 1.2 as compared to pH 7.4. The polymer volume fraction is greatly affected by the composition of polymers and the monomer. A drop is seen in the polymer volume fraction with an increasing concentration of CP, SpS, and AA at both pH 1.2 and 7.4, respectively. This may be correlated with the swelling index of the developed hydrogels. The low and high values of the polymer volume fraction at pH 7.4 and 1.2 indicate the significant swelling and pronounced expansion capability of the formulated hydrogel networks [42].

### 2.4. Thermogravimetric Analysis (TGA)

**Figure 2:**
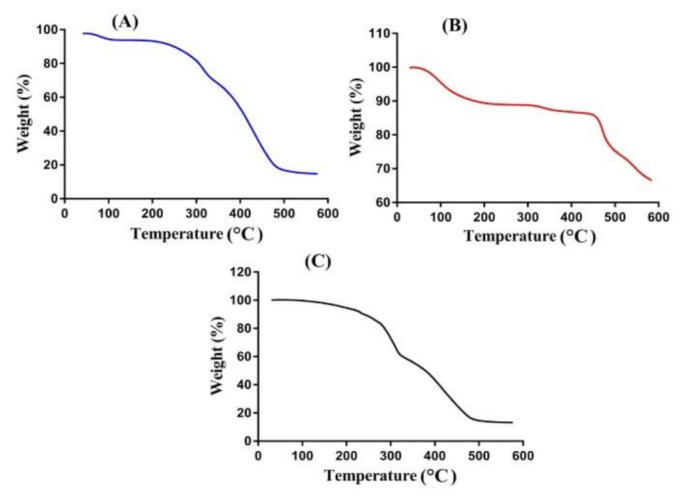
TGA of (**A**) CP, (**B**) SpS, and (**C**) CP/SpScPAA hydrogels.

TGA analysis is conducted for CP, SpS, and CP/SpScPAA hydrogels to evaluate and analyze the thermal stability of unreacted polymers and developed hydrogels. Hence, the TGA analysis of CP (Figure 2A) reveals a 12% weight loss as the temperature approaches 98 °C, representing the moisture loss. A further reduction in weight of 23% is seen as the temperature reaches 331 °C. This weight loss is due to decarboxylation, the preparation of unsaturated structures, and depolymerization of the polymer. The degradation of CP started at 390 °C and continued until paralyzed entirely [43]. Similarly, the TGA analysis of SpS is indicated in Figure 2B. An 8% reduction in the weight of SpS is exhibited as the temperature approaches 248 °C. Further weight loss of 6% is assigned at a temperature of 462 °C, followed by the degradation of SpS with a further increase in temperature [44]. Figure 2C indicates the TGA of CP/SpScPAA hydrogels. Weight loss of 40% is observed initially within the temperature range of 100–310 °C, followed by a further reduction in weight of 42% as the temperature approaches 480 °C, and finally, weight loss of fabricated hydrogels began at 500 °C and continued until complete degradation. The TGA thermogram of unreacted polymers i.e., CP and SpS, and CP/SpScPAA hydrogels reveal that the degradation half-lives of pure polymers i.e., CP (t1/2 = 390 °C) and SpS (t1/2 = 462 °C), are less than the degradation half-life of the developed hydrogels i.e., CP/SpScPAA hydrogels (t1/2 = 500 °C). Hence, the developed hydrogel networks indicate higher thermal stability compared to its unreacted contents. The increase in thermal stability of CP/SpScPAA hydrogels as compared to the unreacted pure polymers could endorse the grafting and cross-linking of hydrogel contents during the polymerization technique [45].

### 2.5. Differential Scanning Calorimetry (DSC)

**Figure 3:**
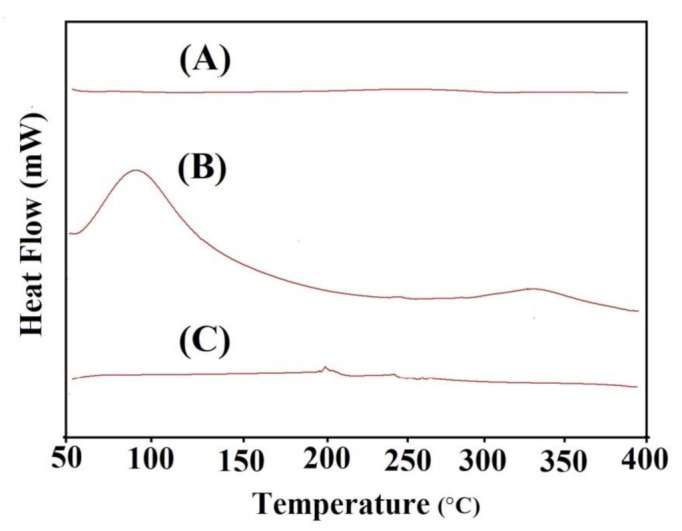
DSC of (**A**) CP, (**B**) SpS, and (**C**) CP/SpScPAA hydrogels.

DSC analysis is performed for pure CP, SpS, and CP/SpScPAA hydrogels as indicated in Figure 3A–C. DSC of CP (Figure 3A) assigns two endothermic peaks at 69 and 229 °C. The first endothermic peak may be assigned to the evaporation of unbound water present in CP while the latter peak may be perceived due to the formation of anhydrides in CP. Furthermore, two exothermic peaks are observed at 90 and 223 °C [46]. Similarly, DSC analysis of SpS and the CP/SpScPAA hydrogel is shown in Figure 3B,C. SpS reveals two minor endothermic peaks at 58 and 260 °C. The glass transition temperature is perceived by an endothermic peak at 58 °C, whereas the thermal degradation of SPS is assigned by an endothermic peak at 260 °C. Two endothermic peaks are indicated by DSC of CP/SpScPAA hydrogels at 198 °C and 247 °C, respectively, followed by moisture loss of the polymers. Similarly, a minor exothermic peak is perceived at 253 °C, followed by the glass phase transition. The discussion shows that the thermal degradation of the fabricated hydrogel networks is perceived within the temperature range of 350–400 °C, indicating higher stability of the fabricated hydrogels compared to CP and SpS [47].

### 2.6. Swelling Studies

**Figure 4:**
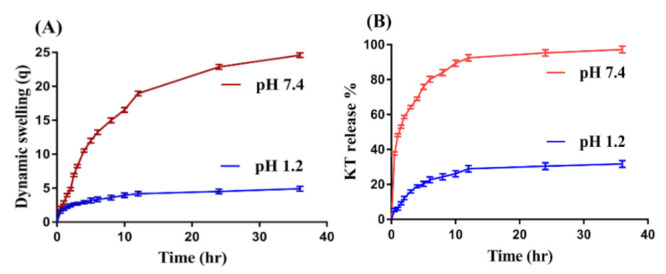
Effect of pH on (**A**) dynamic swelling, and (**B**) KT percent release from CP/SpScPAA hydrogels.

Dynamic swelling is conducted to determine the effects of pH and the composition of hydrogel contents on dynamic swelling of hydrogels in acidic and basic media (pH 1.2 and 7.4) as indicated in Figure 4A and Table 2. pH greatly affects the swelling behavior of the fabricated hydrogels, as less swelling is detected at low pH 1.2 compared to high pH 7.4 (Figure 4A). The main reason for low swelling at pH 1.2 is the protonation of functional groups of CP, SpS, and AA at pH 1.2. CP and AA contain COOH functional groups. Due to the protonation of COOH groups at the lower pH of 1.2, the hydrogel network collapses because strong hydrogen bonding of the COOH groups with counter ions is generated, which leads to shrinkages of the hydrogel network, and hence less swelling is observed at pH 1.2. Similarly, SpS consists of sulfonate groups as same as 2-acrylamido-2-methyl propane sulphonic acid. When the polymerization reaction occurs among CP, SpS, and AA, SpS imparts pH-dependent behavior and exhibits the minimum swelling at pH 1.2 and the maximum at pH 7.4. The protonation of the sulfonate ions of SpS occurs at the lower pH of 1.2, due to which the sulfonate ions are associated and give the maximum strength to the hydrogen bonding, and hence a strong physical interaction is generated among hydrogel constituents. Therefore, low swelling is exhibited at pH 1.2 [48]. Contrary to pH 1.2, maximum swelling is exhibited by hydrogels at pH 7.4. A possible reason is the deprotonation of functional groups of CP, SpS, and AA. CP and AA contain COOH groups, so as the pH of the medium is enhanced from pH 1.2 to 7.4, the deprotonation of COOH groups occurs, which leads to the generation of high charge density. Strong electrostatic repulsive forces are produced among the COOH groups, due to which hydrogen bonding is decreased, and as a result, maximum swelling is exhibited at pH 7.4. Similarly, the ionization/deprotonation of sulfonate groups of SpS occurs at pH 7.4 because charge density is enhanced on hydrogel networks, which leads to strong electrostatic repulsion among its sulfonate groups, and as a result, the developed hydrogels exhibit high swelling at pH 7.4 [49,50,51].

Like pH, the composition of hydrogel contents also influences the dynamic swelling of CP/SpScPAA hydrogels at both pH 1.2 and 7.4. At a constant composition of SpS and AA, the swelling index is increased at both pH 1.2 and 7.4 with an increase in the composition of CP (Table 2). The key point is the increase in the charge density of COOH groups of CP, which leads to maximum swelling especially at pH 7.4, and thus dynamic swelling is increased with an increase in CP composition [52]. Similarly, an increase in SpS composition (Table 2) leads to an increase in the dynamic swelling of hydrogels. A possible cause is the increase in the charge density of sulfonate groups of SpS on the polymeric hydrogel network, which produces strong electrostatic repulsive forces, and hence, an increase in dynamic swelling is exhibited [53]. The pKa value of AA is about 4. Hence, high production of COOH groups is observed as the composition of AA is increased because the greater the quantity of AA, the higher the availability of COOH groups; hence an increase in the hydrophilicity of the hydrogel networks is observed, and therefore greater dynamic swelling will be detected (Table 2), and vice versa [54]. Conclusively, we could demonstrate that the developed hydrogel networks exhibit pH-dependent behavior. Additionally, with the incorporation of CP, the sensitivity and swelling index of the formulated hydrogels is greatly enhanced compared to previously report SpS-based hydrogels.

### 2.7. Scanning Electron Microscopy (SEM)

**Figure 5:**
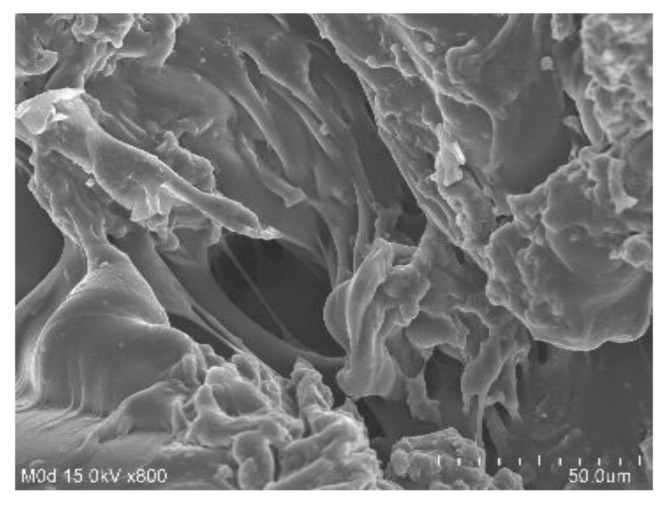
Scanning electron microscopy of CP/SpScPAA hydrogels.

SEM is performed for CP/SpScPAA hydrogels to investigate their surface morphology. A smooth and porous surface is shown by the developed network of hydrogels as shown in Figure 5. The surface of the fabricated hydrogels indicates the strong crosslinking among the hydrogel contents i.e., CP, SpS, and AA. The pores on the surface of the hydrogels provide channels for the penetration of water. The greater the number of pores, the higher the swelling will be, and vice versa [55].

### 2.8. Drug Loading of Hydrogel Samples

A drug loading study was performed for all formulations of the hydrogels to determine the quantity of the drug encapsulated by hydrogels (Table 1). Loading of a drug depends on the swelling of hydrogels, which in turn depends on the porosity of hydrogels. If the porosity of the hydrogels is high, a greater quantity of the drug will be loaded by the developed hydrogels due to high penetration of water through the pore. Conclusively, the greater the porosity, the higher swelling and drug loading will be. Drug loading is increased as the composition of hydrogel contents i.e., CP, SpS, and AA, is increased [56]. The increase in drug loading due to the increase in the composition of CP and AA is because of the deprotonation of COOH groups in a basic medium, which was used for the preparation of the drug solution, and therefore greater swelling and drug loading is exhibited with the increase in the composition of CP and AA. Like CP and AA, swelling is increased as the composition of SpS is enhanced, and as a result, the maximum quantity of the drug is loaded by hydrogels [56,57].

### 2.9. In-Vitro Drug Release Studies

A drug release study is conducted for the purpose of determining the percent drug release from the developed CP/SpScPAA hydrogels at two different pH media i.e., pH 1.2 and 7.4, as indicated in Figure 4B. The maximum percent drug release at pH 7.4 is due to the deprotonation of the functional groups of CP, SpS, and AA. CP and AA contain COOH groups, which exert strong electrostatic repulsive forces generated by high charge density in a basic medium. The strong electrostatic repulsive forces lead to higher swelling, and as a result, the maximum amount of the drug is released. Similarly, the deprotonation of sulfonate groups of SpS occurs at pH 7.4, which also leads to higher swelling and drug release in a basic medium. Unlike at pH 7.4, percent drug release at pH 1.2 is very low due to the protonation of COOH and SO_3_ groups of CP, AA, and SpS. These groups form a conjugate with counter ions, and as a result, strong hydrogen bonding is generated, which cause the hydrogel networks to collapse and hence, a reduction in dynamic swelling and percent drug release is observed [49,58].

Similarly, the effects of the composition of the hydrogel contents on percent drug release at both pH 1.2 and 7.4 are analyzed. A slight decrease in percent drug release is depicted as the composition of CP (Table 2) is increased. A possible reason is the high bulk density and viscosity of the developed system. CP is viscous in nature, and encapsulation of the drug by the hydrogel contents results in an increase in viscosity of the polymeric system and, hence, a decrease in percent drug release is observed [59]. Khan and Zhu et al. (1999) also reported the same results as our study [60]. Unlike CP, an increase in percent drug release is seen as the composition of SpS and AA (Table 2) is increased. The main cause for this increase in drug release is the enhancement in the charge density of both SpS and AA, which increases as the composition of SpS and AA is increased, and vice versa. The discussion indicates that pH-dependent drug release is exhibited by the developed hydrogels as swelling due to the protonation and deprotonation of the functional groups of CP, AA, and SpS [54,61]. Thus, a high percent drug release is shown by the formulated hydrogels compared to previously reported hydrogels of SpS.

### 2.10. Drug Release Kinetics

The release data were fit in all kinetic models for the purpose of understanding the drug release mechanism from the CP/SpScPAA hydrogels. “r” values represent the regression co-efficient. The best kinetic model was chosen on the basis of “r” values close to 1. “r” values of all kinetic models are compared, and first-order kinetics is considered the best model because the “r” values of other kinetic models are found less than that of first-order kinetics, which are almost 1. First-order kinetics has “r” values within the range of 0.9762–0.9965 (Table 3), which shows that CP/SpScPAA hydrogels follow a first-order kinetic model. The type of diffusion is determined by “n” values i.e., the Fickian diffusion mechanism (n = 0.5) and non-Fickian or anomalous (n > 0.5) [62]. “n” values for the developed hydrogels are found within the 0.5017–0.8522 range (Table 3), which means that the developed hydrogels exhibit a non-Fickian diffusion mechanism.

### 2.11. Percent Porosity

**Figure 6:**
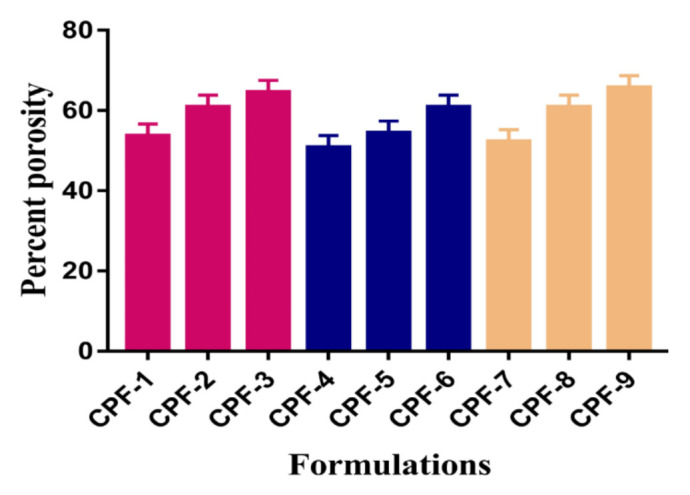
Percent porosity of CP/SpScPAA hydrogels.

Porosity plays an important role in the swelling of hydrogels. The high swelling index of hydrogel formulations is due to the availability of a large number of pores present on the surface of the hydrogels. Hence, the greater the porosity, the higher the dynamic swelling index, and as a result, the drug loading and release will be high. The porosity of developed hydrogels is influenced by the various compositions of polymers and the monomer. Hence, an increase in the percent porosity is observed with the increasing composition of CP and SpS as shown in Figure 6. Similarly, the percent porosity is increased with the increasing AA composition (Figure 6). The reason for this may be the highly viscous nature of the reaction mixture, which prevents bubble leakage from the reaction mixture. Hence, interconnected channels are produced, which leads to an increase in porosity [63].

### 2.12. Powder X-ray Diffraction (PXRD)

**Figure 7:**
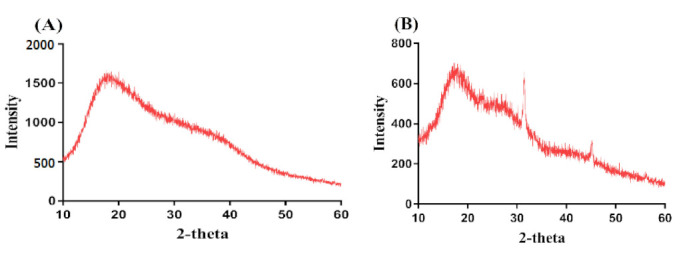

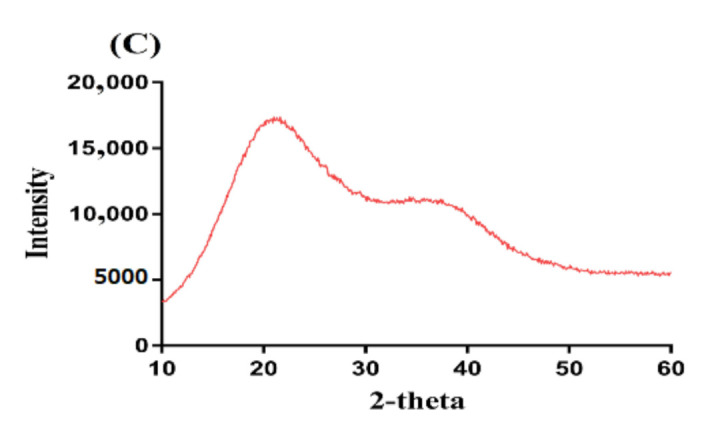
PXRD of (**A**) CP, (**B**) SpS, and (**C**) CP/SpScPAA hydrogels.

The main purpose of PXRD is to evaluate and analyze the crystallinity of the hydrogel constituents i.e., CP, SpS, and the formulation of developed hydrogels. Hence, PXRD is conducted for CP, SpS, and CP/SpScPAA hydrogels as indicated in Figure 7A–C. PXRD of CP and SpS reveals high-intensity crystalline peaks at 2*θ* = 18.13°, 21.24°, 28.41°, and 38.12°, and 2*θ* = 32.40° and 45.28°, respectively (Figure 7A,B. PXRD of CP/SpScPAA hydrogels reveals that the crystalline high-intensity peaks of CP and SpS are reduced or disappeared for the fabricated hydrogels, as indicated in Figure 7C. The disappearance of crystalline peaks of both CP and SpS indicates the formation of strong chemical bonds among the hydrogel contents i.e., CP, SpS, and AA, respectively. The decrease in the crystallinity of the hydrogel contents will help in the swelling of the developed semi-interpenetrating polymer network hydrogels. Greater swelling will lead to high drug loading and release. Chang et al. (2009) prepared cellulose- and alginate-based hydrogels and reported higher amorphous morphology of the fabricated gel [64]. Similarly, Abdullah et al. (2018) reported a reduction in the crystallinity of the hydrogel contents by the fabricated hydrogels [65]. Furthermore, Lee and his coworkers reported a reduction in the crystallinity of the constituents employed for the development of hydrogels [66]. These all support our findings of a higher amorphous morphology of the fabricated hydrogels.

## 3. Conclusions

CP/SpScPAA hydrogels were prepared successfully by the free radical polymerization technique. FTIR analysis confirmed the overlapping of AA over the backbone of CP and SpS. TGA and DSC studies demonstrated higher thermal stability of the fabricated hydrogels as compared to unreacted CP and SpS. SEM revealed a smooth and porous surface of the hydrogels. The high-intensity peaks of CP and SpS disappeared in the developed hydrogels as indicated by PXRD. The pH-responsive nature of the hydrogels was seen by dynamic swelling and percent drug release studies at both low pH of 1.2 and high pH of 7.4, respectively. All formulations of the developed hydrogels followed first-order kinetic modeling. Gel and sol fractions were analyzed by sol-gel analysis and depicted a greater gel fraction with an increase in the composition of CP, SpS, and AA while a decrease in the sol fraction was observed, and vice versa. Similarly, an increase in the percent porosity was observed with the increase in CP, SpS, and AA composition. The results demonstrate that CP/SpScPAA hydrogels have the potential to prolong the release of other NSAIDs in a controlled way.

## 4. Materials and Methods

### 4.1. Materials

Ketorolac tromethamine (KT) was gifted by Jeedimetla, Hyderabad, Telangana, India. Carbopol 934 (CP) was procured from Noveon, Inc, 9911 Brecksville Road, Cleveland, OH, USA, while Sodium poly styrene sulfonate (SpS) was obtained from Alfa Aesar, Ward Hill, MA, USA. Ammonium persulfate (APS) was obtained from Showa, Tokyo, Japan and *N*’,*N*’-methylene bisacrylamide (MBA) was acquired from Alfa Aesar, Lancashire, UK. Acrylic acid (AA) was purchased from Acros (Carlsbad, CA, USA).

### 4.2. Development of CP/SpScPAA Hydrogels

Polymers (CP/SpS) and the monomer (AA) in various concentrations were crosslinked by a cross-linker (MBA) in the presence of an initiator (APS) by the free radical polymerization technique for the development of carbopol934/sodium polystyrene sulfonate-co-poly(acrylic acid) (CP/SpScPAA) hydrogels as shown in Table 4. The accurate weight of CP and SpS was taken separately, and they were then dissolved in distilled water. The CP solution was kept on constant stirring at 50 °C. After this, precise quantities of AA, APS, and MBA were taken. AA was already in liquid form while APS was dissolved in 1 mL of distilled water. MBA is not completely soluble in water, so a mixture of ethanol and water (1:1 *v*/*v*) was used for dissolving MBA completely with constant stirring at 50 °C. The APS solution was added into the SpS solution and stirring continued, then the mixture was added to the CP solution, followed by the dropwise addition of AA. The mixture was kept under constant stirring until all the constituents were mixed properly and then, finally, the MBA solution was added to crosslink the polymers and the monomer on their proper sites. After a few minutes, the formed translucent solution was purged by nitrogen gas to remove any dissolved oxygen. The solution was poured into glass molds and kept in a water bath at 55 °C for initially 2 h, and then enhanced the temperature up to 65 °C for the next 21 h. The prepared gel was cut into 8 mm discs and washed with a mixture of water and ethanol to remove any unreacted contents from the surface of the gels. The discs of gels were kept at room temperature for 24 h initially, and then the discs were placed in a vacuum oven at 40 °C for 7 days for complete dehydration. The dried discs of gels were assessed for further experiments.

### 4.3. Fourier Transform Infrared (FTIR) Spectroscopy 

FTIR spectroscopy was conducted for the purpose of evaluating and analyzing the existence of various functional groups of the drug and constituents, the development of hydrogel network formation, the degree of cross-linking, and structural modifications that happen during the polymerization reaction. Nicolet 380 FTIR (Thermo Fisher Scientific, Ishioka, Japan) with attenuated total reflectance (ATR) technology was used for the analysis of the drug, pure unreacted formulations components, and hydrogel formulations within FTIR spectra of 4000–500 cm^−1^ [67].

### 4.4. Sol-Gel Analysis

Sol-gel analysis was performed to evaluate and measure the quantity of reactants consumed in the preparation of hydrogels. Hence, the Soxhlet extraction process was conducted for all formulations of developed hydrogels. A weighed amount of dried discs of the hydrogels (A_1_) was accurately taken and immersed in a round-bottom flask (containing deionized distilled water) connected with a condenser at 85 °C for 12 h. After 12 h, the hydrogel disc was extracted and placed in the vacuum oven at 40 °C until a constant weight (A_2_) was obtained [68]. The following equations were used for sol-gel analysis:

(1) $$\mathrm{Sol}\;\mathrm{fraction}\;\%=\frac{{\;A}_{1}-{\;A}_{2}}{{\;A}_{2}}\times 100\;$$

(2) Gel fraction =100−Sol fraction 

A_1_ is the initial weight of the hydrogels and A_2_ is the final weight of the dried hydrogels.

### 4.5. Polymer Volume Fraction

The polymer fraction was evaluated in the swelled state at both pH 1.2 and 7.4 for all formulations of fabricated hydrogels. It is represented by V2. The polymer volume fraction was determined using the equilibrium volume swelling (Veq) data [42]. Hence, the following equation was used:

(3) $$V2,\;s=\frac{1}{\mathrm{Veq}}$$

### 4.6. Thermogravimetric Analysis (TGA) 

TGA was carried out for the purpose of measuring the quantity and rate of change in the weight of the sample of unreacted polymers and developed hydrogels as a function of temperature in a controlled setting. The PerkinElmer Simultaneous Thermal Analyzer STA 8000 (PerkinElmer, Waltham, MA, USA) was used for TGA analysis. Powdered samples of 0.5–5 mg weight were placed in a sample pan for analysis within the temperature range of 40–600 °C, under a heating rate of 20 °C/min. The flow rate of nitrogen gas was kept at 20 mL/min throughout the experiment [69]. 

### 4.7. Differential Scanning Calorimetry (DSC)

DSC is a thermal analysis technique conducted to analyze the heat flow rate of a material with an increase in temperature while using PerkinElmer DSC 4000 (Waltham, MA, USA). This technique could be used to analyze the quantity of energy absorbed or released by a material upon heating or cooling. In total, 0.5–3.5 mg samples of unreacted polymers and hydrogel formulations were placed in a sample pan and the samples were scanned at a temperature of 50–400 °C with a heating rate of 20 °C /min. The stream rate of nitrogen gas was kept 20 mL/min [70].

### 4.8. Swelling Studies

Dynamic swelling studies were carried out at different pH buffer solutions i.e., simulated gastric fluid (pH 1.2) and simulated intestinal fluid (pH 7.4), in order to determine the effect of pH and compositions of hydrogel contents on the swelling behavior of developed hydrogels. Hence, a precise amount of hydrogel discs was immersed in 100 mL buffer solutions of pH 1.2 and 7.4 at 37 °C. At predetermined intervals, the swollen disc was removed from the respective buffer solution, blotted with filter paper to remove the extra solvent, and then weighed again. The swollen hydrogel disc was immersed back in the respective buffer medium. This process was continued until an equilibrium weight of the hydrogel disc was attained [71]. The obtained data were measured in triplicate. The following equations were used for calculating the dynamic swelling and %ge swelling ratio:

(4) $$\;\left(q\right)=\frac{{\;L}_{2}}{L_{1}}\;$$

where q is the dynamic swelling, L_1_ is the initial weight of hydrogel discs before swelling, and L_2_ is the final weight of the swelled hydrogel discs at time t.

(5) $$\mathrm{SR}\%=\frac{{\;N}_{1}-{\;N}_{2}}{N_{2}}\times 100\;$$

where N_1_ is the weight of the swollen hydrogels discs, while N_2_ is the weight of the dried hydrogel discs.

### 4.9. Scanning Electron Microscopy (SEM)

SEM (JSM-6490A, Tokyo, Japan) was used for the structural analysis of developed hydrogels to examine their surface morphology. A dried hydrogel disc was crushed into the required particle size, which was then fixed on an aluminum stub with the help of double adhesive tape. A gold sputter coater was employed for the coating of gold on stubs under an argon atmosphere. Photomicrographs were used for surface morphology analysis of the developed hydrogels [72].

### 4.10. Drug Loading of Hydrogel 

The swelling diffusion method was used for the loading of KT by all formulations of the fabricated hydrogels. A 1% KT solution was prepared in a phosphate buffer of pH 7.4 for the loading of the drug. Accurately weighed dried hydrogel discs were immersed in a 1% KT solution for 72 h at room temperature. The discs were kept in the drug solution for 72 h until an equilibrium weight was achieved at room temperature. After 72 h, the discs were removed, washed by water, and placed in a vacuum oven at 40 °C until a constant weight for the hydrogel disc was obtained [73].

The weight method was used for calculating the drug loaded by the hydrogel disc. The weight of the dried hydrogel discs before immersion in the drug solution was subtracted from the weight of the dried hydrogel discs achieved after soaking them in the drug solution [7]. The following equation was used for calculating the drug loading:

(6) $$\mathrm{Amount}\;\mathrm{of}\;\mathrm{drug}\;=\;\;W_{m}\;-\;W_{n}$$

where W_m_ is the weight of loaded discs of the hydrogel and W_n_ is the weight of the unloaded discs of the hydrogel.

### 4.11. In Vitro Drug Release Studies

In vitro drug release studies were carried out for the purpose of analyzing and investigating the percent release of the drug from the developed hydrogels at different buffer solutions i.e., simulated gastric fluid (pH 1.2) and simulated intestinal fluid (pH 7.4), in order to determine the release of the drug at low pH (simulating stomach conditions) and high pH (simulating a basic condition). In total, a 900 mL buffer solution of pH 1.2 and 7.4 was used for dissolution studies using USP dissolution apparatus-II (Sr8plus Dissolution Test Station) at 37 ± 0.5 °C and 50 rpm. The weighed, dried, loaded hydrogel discs were immersed in a buffer solution of 900 mL in a dissolution apparatus. Aliquots of 5 mL of the samples were withdrawn at predetermined intervals of time and a fresh medium of the same volume was added to maintain the same sink conditions. The collected samples were analyzed by using a UV–vis-spectrophotometer (U-5100,3J2-0014, Tokyo, Japan) at the λ max value of 280 nm [74,75].

### 4.12. Drug Release Kinetics

Various kinetic models such as zero order, first order, Higuchi, and Korsmeyer–Peppas models were assessed by fitting the obtained in vitro release data to determine the drug release mechanism from the fabricated crosslinked hydrogels [6].

### 4.13. Percent Porosity

The percent porosity of all formulations of the fabricated hydrogels was evaluated by the solvent replacement technique. A precise quantity of dried hydrogel discs (*P*_1_) was immersed in absolute ethanol (purity > 99.9%) for 5 days to achieve equilibrium swelling. After that, hydrogels discs were removed, wiped with filter paper to remove any excess solvent, and weighed again (*P*_2_). Similarly, the thickness and diameter of the hydrogel discs were measured [76]. Percent porosity was determined by the following equation.

(7) $$\mathrm{Porosity}\;\mathrm{percentage}\;(\%)\;=\;\;\frac{P-P_{1}}{\rho V}\times \;100$$

*ρ* indicates the density of absolute ethanol, while *V* shows the volume of the hydrogel after swelling. 

### 4.14. Powder X-ray Diffraction (PXRD) Analysis

PXRD (XRD-6000 SHIMADZU, Tokyo, Japan) was performed to understand the crystallinity of polymers and the developed hydrogels. The angle of diffraction was maintained within a range of 10°–60° at a rate of 2° 2θ/min [77].

## Figures and Tables

**Table 1:** Sol-gel analysis, drug loading, and polymer volume fraction of CP/SpScPAA hydrogels.

F. Code	Sol Fraction %	Gel Fration %	Drug Loaded (mg) /400 mg of Dry Gel	Polymer Volume Fration	
				pH 1.2	pH 7.4
CPF-1	10.12 ± 0.08	89.88 ± 0.13	204.96 ± 1.08	0.229	0.053
CPF-2	08.67 ± 0.10	91.93 ± 0.09	232.30 ± 0.98	0.217	0.043
CPF-3	06.32 ± 0.07	93.68 ± 0.11	245.43 ± 1.13	0.207	0.040
CPF-4	11.03 ± 0.11	88.97 ± 0.08	180.64 ± 1.18	0.232	0.057
CPF-5	09.21 ± 0.13	90.79 ± 0.05	199.31 ± 1.14	0.225	0.049
CPF-6	08.67 ± 0.10	91.93 ± 0.09	232.30 ± 0.98	0.217	0.043
CPF-7	09.92 ± 0.08	90.08± 0.12	196.85 ± 1.21	0.229	0.050
CPF-8	08.67 ± 0.10	91.93 ± 0.09	232.30 ± 0.98	0.217	0.043
CPF-9	06.89 ± 0.08	93.11 ± 0.13	248.81 ± 1.20	0.204	0.041

**Table 2:** Dynamic swelling and percent drug release of CP/SpScPAA hydrogels.

F. Code	Dynamic Swelling Up to 36 h		Percent Drug Release Up to 36 h	
	pH 1.2	pH 7.4	pH 1.2	pH 7.4
CPF-1	4.35 ± 0.24	18.73 ± 0.31	30.03 ± 0.74	96.35 ± 0.91
CPF-2	4.60 ± 0.12	22.82 ± 0.26	28.62 ± 0.98	94.80 ± 0.78
CPF-3	4.82 ± 0.08	24.70 ± 0.33	27.12 ± 1.20	91.48 ± 1.28
CPF-4	4.31 ± 0.13	17.51 ± 0.28	23.25 ± 1.53	88.61 ± 1.23
CPF-5	4.43 ± 0.10	20.40 ± 0.13	26.37 ± 1.29	91.90 ± 1.40
CPF-6	4.60 ± 0.12	22.82 ± 0.26	28.62 ± 0.98	94.80 ± 0.78
CPF-7	4.35 ± 0.11	19.63 ± 0.31	25.90 ± 1.39	90.48 ± 1.21
CPF-8	4.60 ± 0.12	22.82 ± 0.26	28.62 ± 0.98	94.80 ± 0.78
CPF-9	4.90 ± 0.19	24.60 ± 0.19	30.73 ± 1.27	97.22 ± 1.61

**Table 3:** Kinetic modeling release of drug from CP/SpScPAA hydrogels.

F. Code	Zero Order r^2^	First Order r^2^	Higuchi r^2^	Korsmeyer-Peppas	
				r^2^	n
CPF-1	0.9555	0.9925	0.9919	0.9897	0.6284
CPF-2	0.9903	0.9910	0.9639	0.9909	0.6219
CPF-3	0.9915	0.9932	0.9796	0.9901	0.6220
CPF-4	0.9646	0.9762	0.9812	0.9840	0.6626
CPF-5	0.9804	0.9869	0.9829	0.9880	0.6542
CPF-6	0.9903	0.9910	0.9639	0.9909	0.6219
CPF-7	0.9930	0.9965	0.9794	0.9962	0.8522
CPF-8	0.9903	0.9910	0.9639	0.9909	0.6219
CPF-9	0.9219	0.9870	0.9880	0.9752	0.5017

**Table 4:** Feed ratio scheme for formulation of CP/SpScPAA hydrogels.

F. Code	Polymer (CP) g/100 g	Polymer (SpS) g/100 g	Monomer (AA) g/100 g	Initiator (APS) g/100 g	Cross-Linker (MBA) g/100 g
CPF-1	0.25	1.00	25	0.5	0.5
CPF-2	0.50	1.00	25	0.5	0.5
CPF-3	0.75	1.00	25	0.5	0.5
CPF-4	0.50	0.50	25	0.5	0.5
CPF-5	0.50	0.75	25	0.5	0.5
CPF-6	0.50	1.00	25	0.5	0.5
CPF-7	0.50	1.00	20	0.5	0.5
CPF-8	0.50	1.00	25	0.5	0.5
CPF-9	0.50	1.00	30	0.5	0.5
